# Suppression of Aldosterone Synthesis and Secretion by Ca^2+^ Channel Antagonists

**DOI:** 10.1155/2012/519467

**Published:** 2012-10-11

**Authors:** Keiichi Ikeda, Tsuyoshi Isaka, Kouki Fujioka, Yoshinobu Manome, Katsuyoshi Tojo

**Affiliations:** ^1^Department of Molecular Cell Biology, Institute of DNA Medicine, Research Center for Medical Sciences, The Jikei University School of Medicine, 3-25-8 Nishishinbashi, Minato-ku, Tokyo 105-8461, Japan; ^2^Division of Diabetes and Endocrinology, Department of Internal Medicine, The Jikei University School of Medicine, 3-25-8 Nishishinbashi, Minato-ku, Tokyo 105-8461, Japan

## Abstract

Aldosterone, a specific mineralocorticoid receptor (MR) agonist and a key player in the development of hypertension, is synthesized as a final product of renin-angiotensin-aldosterone system. Hypertension can be generally treated by negating the effects of angiotensin II through the use of angiotensin-converting enzyme inhibitors (ACE-Is) or angiotensin II type 1 receptor antagonists (ARBs). However, the efficacy of angiotensin II blockade by such drugs is sometimes diminished by the so-called “aldosterone breakthrough” effect, by which ACE-Is or ARBs (renin-angiotensin system (RAS) inhibitors) gradually lose their effectiveness against hypertension due to the overproduction of aldosterone, known as primary aldosteronism. Although MR antagonists are used to antagonize the effects of aldosterone, these drugs may, however, give rise to life-threatening adverse actions, such as hyperkalemia, particularly when used in conjunction with RAS inhibitors. Recently, several groups have reported that some dihydropyridine Ca^2+^ channel blockers (CCBs) have inhibitory actions on aldosterone production in *in vitro* and in the clinical setting. Therefore, the use of such dihydropyridine CCBs to treat aldosterone-related hypertension may prove beneficial to circumvent such therapeutic problems. In this paper, we discuss the mechanism of action of CCBs on aldosterone production and clinical perspectives for CCB use to inhibit MR activity in hypertensive patients.

## 1. Introduction

Aldosterone is an endogenous mineralocorticoid receptor (MR) agonist synthesized in the adrenal glomerular layer as a final product of the renin-angiotensin-aldosterone system (RAAS); it is strongly involved in the development of hypertension due to excessive sodium retention. It has been reported that suppression of the renin-angiotensin system (RAS) by angiotensin-converting enzyme inhibitors (ACE-Is) and angiotensin II type 1 receptor blockers (ARBs) provides an effective treatment against cardiovascular diseases such as hypertension and cardiac failure [1, 2]. Several studies have also revealed that the blockade of MR by an MR antagonist (MRA), such as spironolactone or eplerenone, offers an effective approach to treat cardiac disease, especially cardiac failure [3–6]. These facts indicate that RAAS may contribute to the underlying mechanisms of cardiac diseases for which its control may play a critical role in ameliorating the effectiveness of treatments [7]. Although the blockade of RAS by ACE-Is or ARBs (RAS inhibitors) may be effective, the long-term treatment of hypertension by drugs classified as such often results in a diminished efficacy owing to the inadequate suppression of aldosterone synthesis. This phenomenon is known as “aldosterone breakthrough” [8, 9]. An effective approach may therefore be to use an MRA in addition to RAS inhibitors to avoid such deterioration of the ACE-I/ARB efficacy due to aldosterone breakthrough. To this extent, however, MRA use has been associated with an increased risk of fatal hyperkalemia, and the concomitant use of MRAs with RAS inhibitors may have synergistic effects, potentiating the risk for hyperkalemia [10]. In addition, aldosterone-related hypertension may also be caused by autonomous aldosterone secretion, such as primary hyperaldosteronism, which is often associated with severe hypertension and obesity [11]. These findings indicate that it is necessary that the suppression of aldosterone production be considered as an alternative choice to control blood pressure. Recently, several groups have reported that Ca^2+^ channel blockers (CCBs), which are another class of antihypertensive agent widely used to control blood pressure, may have inhibitory actions on aldosterone synthesis. Here, we provide an overview of the effects of CCBs on the production of aldosterone and discuss clinical perspectives of their use to curb aldosterone production.

## 2. Overview of Steroid Biosynthesis in Adrenal Cells

**Figure 1:**
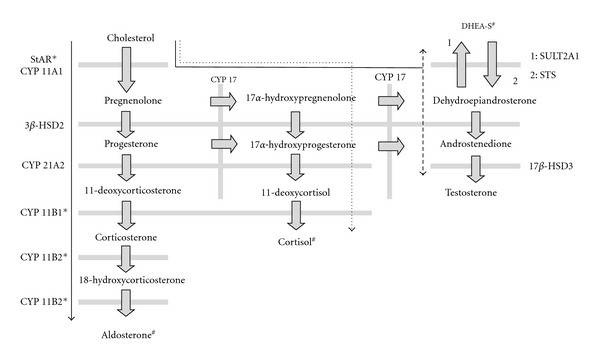
Overview of steroid biosynthesis in adrenal cells. Asterisks (*) and the symbol (^#^) indicate enzymes and the adrenal steroid that may be modulated by dihydropyridine CCBs. Solid line: steroid biosynthesis in cells of the zona glomerulosa; dotted line: steroid biosynthesis in cells of the zona fasciculate; broken line: steroid biosynthesis in cells of the zona reticularis.

A brief summary of steroid biosynthesis in human adrenal cells is provided here for the convenience of the reader. The biosynthetic pathways of adrenal steroids are summarized in Figure 1 [12, 13]. Briefly, steroid biosynthesis is initiated by steroidogenic acute regulatory protein (StAR), which transports cholesterol into the mitochondria. The side chain of cholesterol is then cleaved by cytochrome P450 side chain cleavage enzyme (CYP11A1) to produce pregnenolone. In the zona glomerulosa of the adrenal gland (solid line in Figure 1), which does not express cytochrome P450 17*α*-hydroxylase/17,20 lyase (CYP17), pregnenolone is converted to progesterone by 3*β*-hydroxysteroid dehydrogenase type 2 (3*β*-HSD2). CYP17, which is expressed by cells of the zona fasciculata (dotted line in Figure 1) and the zona reticularis (broken line in Figure 1), catalyzes the conversion of pregnenolone and progesterone to 17*α*-hydroxypregnenolone and 17*α*-hydroxyprogesterone, respectively. Via a different pathway, progesterone is catalyzed to 11-deoxycorticosterone (DOC), and 17*α*-hydroxyprogesterone to 11-deoxycortisol, respectively, by hydroxylation with steroid 21-hydroxylase (CYP21A2). Corticosterone is generated from DOC by 11*β*-hydroxylase (CYP11B1), which also generates cortisol from 11-deoxycortisol in zona fasciculata cells, and, in turn, aldosterone is generated from 18-hydroxycorticosterone by CYP11B2, which is also known as aldosterone synthetase. The regulation of CYP11B2 is mediated by Ca^2+^-sensitive manner through mechanisms involving calmodulin and calmodulin-dependent kinases, and the 11*β*-hydroxylase activity is also stimulated by Ca^2+^ [14, 15]. The previous experiments revealed that angiotensin II-induced aldosterone synthesis is involved in activation of the low voltage-activated T-type Ca^2+^ channel [16, 17], and the expression of CYP11B2 mRNA was suppressed by some dihydropyridine CCBs, which can inhibit the T-type Ca^2+^ channel [18–20]. In the adrenal cells of the zona reticularis, 17*α*-hydroxypregnenolone and 17*α*-hydroxyprogesterone are also catalyzed to dehydroepiandrosterone (DHEA) and androstenedione, respectively. The DHEA is further sulfated to DHEA-sulfate (DHEA-S) by sulfotransferase (SULT2A1) and reversely sulfated from DHEA-S by steroid sulfatase (STS). Androstenedione on the other hand is converted to testosterone by 17*β*-hydroxysteroid dehydrogenase type 3 (17*β*-HSD3).

Both the expression of StAR at mRNA and protein levels and its activity were shown to be increased by nifedipine and efonidipine in MA-10 mouse Leydig cells and NCI-H295R human adrenocortical carcinoma cells, but decreased by amlodipine, azelnidipine, or *R*(-)-efonidipine [21, 22]. Likewise, CYP11B1 and CYP11B2 are decreased by azelnidipine, benidipine, and efonidipine (* in Figure 1) in NCI-H295R human adrenocortical carcinoma cells [18–20], while efonidipine increases DHEA-S production in NCI-H295R human adrenocortical carcinoma cells probably as a result of increased StAR expression (^#^ in Figure 1) [22]. The reported actions of dihydropyridine CCBs on the expression of steroidogenic enzymes in *in vitro* studies are summarized in Tables 1 and 2 [18–25].

## 3. Actions of Dihydropyridine CCBs on Adrenal Steroid Synthesis

A previous study reported that a specific step in the steroidogenic pathway may be directly linked to agonist-induced increases in the cytosolic free Ca^2+^ concentration in intact isolated zona glomerulosa cells [26]. Stimulation by angiotensin II or exposure to high extracellular potassium (which depolarized the cell membrane) elevates the intracellular Ca^2+^ [27] and induces aldosterone production. This increase in intracellular Ca^2+^ can be blocked by the L-type CCB nifedipine, though not completely in the case of the angiotensin II-induced intracellular Ca^2+^ elevation [27], with a concomitant decrease in aldosterone production [28]. In addition, even verapamil and diltiazem, which are nondihydropyridine L-type Ca^2+^ channel antagonists, exerted inhibitory actions on aldosterone production in rat's adrenal glands [29]. These findings indicate that the production of aldosterone may be stimulated by processes other than by Ca^2+^ influx through the L-type calcium channel. For example, other studies on adrenal glomerulosa cells, in which T-type Ca^2+^ channel-specific antagonists (mibefradil, tetrandrine, and Ni^2+^) were used, reported involvement of the T-type Ca^2+^ channel in aldosterone production in addition to the L-type Ca^2+^ channel [28, 30–32]. These results indicate that adrenal aldosterone production requires the activation of T-type Ca^2+^ channels. Thereafter, it was reported that a newly developed dihydropyridine CCB, efonidipine, which has inhibitory properties for both L- and T-type Ca^2+^ channels [33], suppressed aldosterone and cortisol production more potently than nifedipine by reducing the expression of CYP11B1 and CYP11B2 [18, 20]. Interestingly, however, when used on NCI-H295R human adrenocortical carcinoma cells, efonidipine increased the expression of StAR mRNA and protein, possibly resulting in the increased production of DHEA-S [22]. It has also been reported that azelnidipine and benidipine, as well as efonidipine, have inhibitory actions on adrenal aldosterone production by decreasing the expression of CYP11B1 and CYP11B2 more potently than nifedipine [19, 20], and cilnidipine suppressed angiotensin II-induced CYP11B2 mRNA expression, but not CYP11B1. Moreover, while azelnidipine, benidipine, and efonidipine have inhibitory properties against T-type Ca^2+^ channels (*α*1H and *α*1G), nifedipine has little effect on these T-type Ca^2+^ channel subtypes [24], indicating that the effects of CCBs on the T-type Ca^2+^ channel, in addition to that on the L-type Ca^2+^ channel, may be involved in their inhibitory actions on aldosterone production. Recently, another group reported that cilnidipine, which has little or no effect on T-type Ca^2+^ channels [24] but significant inhibitory actions against an N-type Ca^2+^ channel [34], also suppressed aldosterone production in NCI-H295R human adrenocortical carcinoma cells [23]. The N-type Ca^2+^ channel may be involved in aldosterone production, because *ω*-conotoxin GIVA, a specific N-type Ca^2+^ channel blocker, significantly suppressed aldosterone and cortisol secretions in NCI-H295R human adrenocortical carcinoma cells without significantly influencing CYP11B2 or CYP11B1 mRNA expression [23].

Given that aldosterone is a key factor in cardiac pathological stress, promoting processes such as fibrosis and oxidative stress [7], it is important to evaluate the effects of above-mentioned CCBs on aldosterone production in *in vivo* animal preparations and in patients with cardiovascular diseases. In this way, *in vivo* studies with benidipine and cilnidipine were found to reduce the plasma aldosterone concentration (PAC) in stroke-prone spontaneously hypertensive rats [35], in the ischemia reperfusion mouse model [36], and in male SHR/Izm rats [37]. In clinical studies, azelnidipine, benidipine, and efonidipine were shown to exert suppressive actions on PAC in hypertensive patients with type 2 diabetes mellitus [38], in patients with mild-to-moderate stage chronic kidney disease with albuminuria [39], in patients with chronic glomerulonephritis [40], and in patients with essential hypertension [41].

Activation of MR by aldosterone is one of the important causes of arterial hypertension, and, due to the extrarenal effects of aldosterone, such as cardiac fibrosis and vascular inflammation [42], it is very important to antagonize the MR activities in such patients. Dihydropyridine CCBs are widely recommended in the treatment of hypertension by several guidelines [43–47] and may have more therapeutic potential in combination with antagonists for RAS [48–50]. Combination therapies with RAS inhibitors and CCBs are well-tolerated in hypertensive patients [51–54], indicating that CCBs are the potentially important candidates as a concomitant drug with RAS inhibitors. Also, because monotherapy by RAS inhibitors for hypertension may often cause diminished efficacy of treatment, so-called “aldosterone breakthrough [8, 9],” treatment of hypertensive patients with RAS inhibitor alone is often required to add another antihypertensive drug to avoid aldosterone breakthrough. MRAs are often considered for such purpose, but concomitant usage of MRAs with RAS inhibitors increases incidence of hyperkalemia, which is one of the life-threatening adverse effects [10]. Furthermore, recent data suggest that primary aldosteronism is present in approximately 10% of hypertensive patients [55], indicating that the suppressive property of adrenal aldosterone production without severe adverse effects may be a key element in treatment of hypertension. Therefore, CCBs, which suppress adrenal aldosterone production and have tolerable property in concomitant usage with RAS inhibitors, may be another useful choice to overcome aldosterone breakthrough and aldosterone-related hypertension without intolerable adverse effects, such as severe hyperkalemia. Therefore, taken together with the recently reported data concerning the antagonistic properties of CCBs against MR activity [56, 57], such dihydropyridine CCBs may act as a new class of MRAs providing a therapeutic advantage for the treatment of aldosterone-related hypertensive patients.

## 4. Conclusion

Recent studies have revealed that dihydropyridine CCBs, such as azelnidipine, benidipine, cilnidipine, efonidipine, and nifedipine, have inhibitory actions on adrenal aldosterone biosynthesis* in vitro*. Some studies have also shown that plasma aldosterone levels are decreased in the patients prescribed such dihydropyridine CCBs. Based on accumulating evidence from *in vitro *and clinical studies of the actions of these drugs on aldosterone production, the clinical use of dihydropyridine CCBs may provide therapeutic advantages to combat aldosterone-related hypertension in affected patients.

## Figures and Tables

**Table 1:** The effects of dihydropyridine CCBs on dbcAMP- or KCl-induced expression of steroidogenic enzymes.

Dihydropyridine CCB	Type of affecting Ca^2+^ channels	StAR	CYP11A1	3*β*HSD2	CYP21A2	CYP11B1	CYP11B2	STS	SULT2A1
Amlodipine	L, T	→	→	→	→			→	→
Azelnidipine	L, T	→	→	→	→	↓	↓	→	→
Benidipine	L, T					↓	↓		
Efonidipine	L, T	↑	→	→	→	↓	↓	→	→
Isradipine	L, T	↑							
Nifedipine	L	↑	→	→	→	↓	↓	→	→
Nitrendipine	L					↓	↓		

dbcAMP: N^6^, 2′-O-dibutyryladenosine 3′, and 5′-cyclic monophosphate.

**Table 2:** The effects of dihydropyridine CCBs on angiotensin II-induced expression of steroidogenic enzymes.

Dihydropyridine CCB	Type of affecting Ca^2+^ channels	StAR	CYP11B1	CYP11B2
Amlodipine	L, T	→		
Azelnidipine	L, T	↓	↓	↓
Benidipine	L, T		↓	↓
Cilnidipine	L, N		→	↓
Efonidipine	L, T	↑	↓	↓
Nifedipine	L	→	↓	↓
Nitrendipine	L		→	→
